# Differentiation of Benign From Malignant Parotid Gland Tumors Using Conventional MRI Based on Radiomics Nomogram

**DOI:** 10.3389/fonc.2022.937050

**Published:** 2022-07-11

**Authors:** Jinbo Qi, Ankang Gao, Xiaoyue Ma, Yang Song, Guohua zhao, Jie Bai, Eryuan Gao, Kai Zhao, Baohong Wen, Yong Zhang, Jingliang Cheng

**Affiliations:** ^1^ Department of MRI, The First Affiliated Hospital of Zhengzhou University, Zhengzhou, China; ^2^ Magnetic Resonance Scientific Marketing, Siemens Healthineers Ltd., Shanghai, China

**Keywords:** parotid gland tumor, radiomics, magnetic resonance imaging, nomogram, pleomorphic adenoma, Warthin tumor

## Abstract

**Objectives:**

We aimed to develop and validate radiomic nomograms to allow preoperative differentiation between benign- and malignant parotid gland tumors (BPGT and MPGT, respectively), as well as between pleomorphic adenomas (PAs) and Warthin tumors (WTs).

**Materials and Methods:**

This retrospective study enrolled 183 parotid gland tumors (68 PAs, 62 WTs, and 53 MPGTs) and divided them into training (n = 128) and testing (n = 55) cohorts. In total, 2553 radiomics features were extracted from fat-saturated T2-weighted images, apparent diffusion coefficient maps, and contrast-enhanced T1-weighted images to construct single-, double-, and multi-sequence combined radiomics models, respectively. The radiomics score (Rad-score) was calculated using the best radiomics model and clinical features to develop the radiomics nomogram. The receiver operating characteristic curve and area under the curve (AUC) were used to assess these models, and their performances were compared using DeLong’s test. Calibration curves and decision curve analysis were used to assess the clinical usefulness of these models.

**Results:**

The multi-sequence combined radiomics model exhibited better differentiation performance (BPGT *vs*. MPGT, AUC=0.863; PA *vs*. MPGT, AUC=0.929; WT *vs*. MPGT, AUC=0.825; PA *vs*. WT, AUC=0.927) than the single- and double sequence radiomics models. The nomogram based on the multi-sequence combined radiomics model and clinical features attained an improved classification performance (BPGT *vs*. MPGT, AUC=0.907; PA *vs*. MPGT, AUC=0.961; WT *vs*. MPGT, AUC=0.879; PA *vs*. WT, AUC=0.967).

**Conclusions:**

Radiomics nomogram yielded excellent diagnostic performance in differentiating BPGT from MPGT, PA from MPGT, and PA from WT.

## Introduction

Parotid gland tumors account for approximately 80% of salivary gland tumors (1). Approximately 20% of tumors that arise in the parotid gland are malignant, which is markedly lower than the incidence of benign parotid gland tumors (BPGT) (1, 2). Mucoepidermoid carcinoma is the most common malignant parotid gland tumor (MPGT) (2, 3), whereas pleomorphic adenoma (PA) is the most common BPGT, followed by Warthin’s tumor (WT) (2). To account for differences in surgical methods and prognosis, one must differentiate BPGT from MPGT. Accurate preoperative differentiation of PA from WT also influences the surgical method, as PAs have a high incidence of recurrence and malignant transformation (4, 5).

Fine-needle aspiration (FNA) biopsy is a common preoperative examination method for parotid neoplasms. However, FNA has some disadvantages, such as high rates of insufficient diagnostic aspirations and the risk of facial nerve palsy (6, 7). Magnetic resonance imaging (MRI) has the characteristics of noninvasive multidirectional imaging and high soft-tissue resolution, which is important when evaluating parotid gland tumors. In MRI sequences, fat-saturated T2-weighted imaging (FS-T2WI) can provide anatomical information on the tumor, apparent diffusion coefficients (ADCs) from diffusion-weighted imaging (DWI) can be used to diagnose disease severity by analyzing the diffusion motion of local water molecules, and contrast-enhanced T1-weighted imaging (CE-T1WI) provides information on tumor blood supply (8–10). Morphological features of the tumor can help differentiate between benign and malignant tumors, including anatomical position (superficial *vs*. deep), margins, heterogeneous appearance, and infiltration of surrounding tissue, which can be found on MRI. However, identifying these signs requires high reader expertise and a high workload.

Radiomics can extract high-dimensional features from medical images, provide more comprehensive tumor descriptions, and improve diagnostic performance and clinical prediction (11–13). It has been widely applied in tumor research for clinical diagnosis, prognostic assessment, and gene prediction (9, 14, 15). Piludu et al. (16) recently indicated that radiomics-based ADC maps and T2WI have good diagnostic performance when differentiating parotid lesions. Zheng et al. (17) reported that a radiomics nomogram differentiated BPGT from MPGT when T1-weighted imaging (T1WI) and FS-T2WI were used. In addition, Shao et al. (18) showed that a DWI-based triple-classification radiomics model has predictive value in distinguishing PA, WT, and MPGT. However, the above studies lacked information directly related to local blood supply in tumors, which is valuable for differentiating BPGT from MPGT.

Therefore, this study constructed single-, double-, and multi-sequence combined radiomics models based on FS-T2WI, ADC, and CE-T1WI, and compared the diagnostic performance of these models to construct a radiomics nomogram to preoperatively differentiate BPGT from MPGT and differentiate PA and WT from BPGT.

## Materials and Methods

### Patients

**Figure 1:**
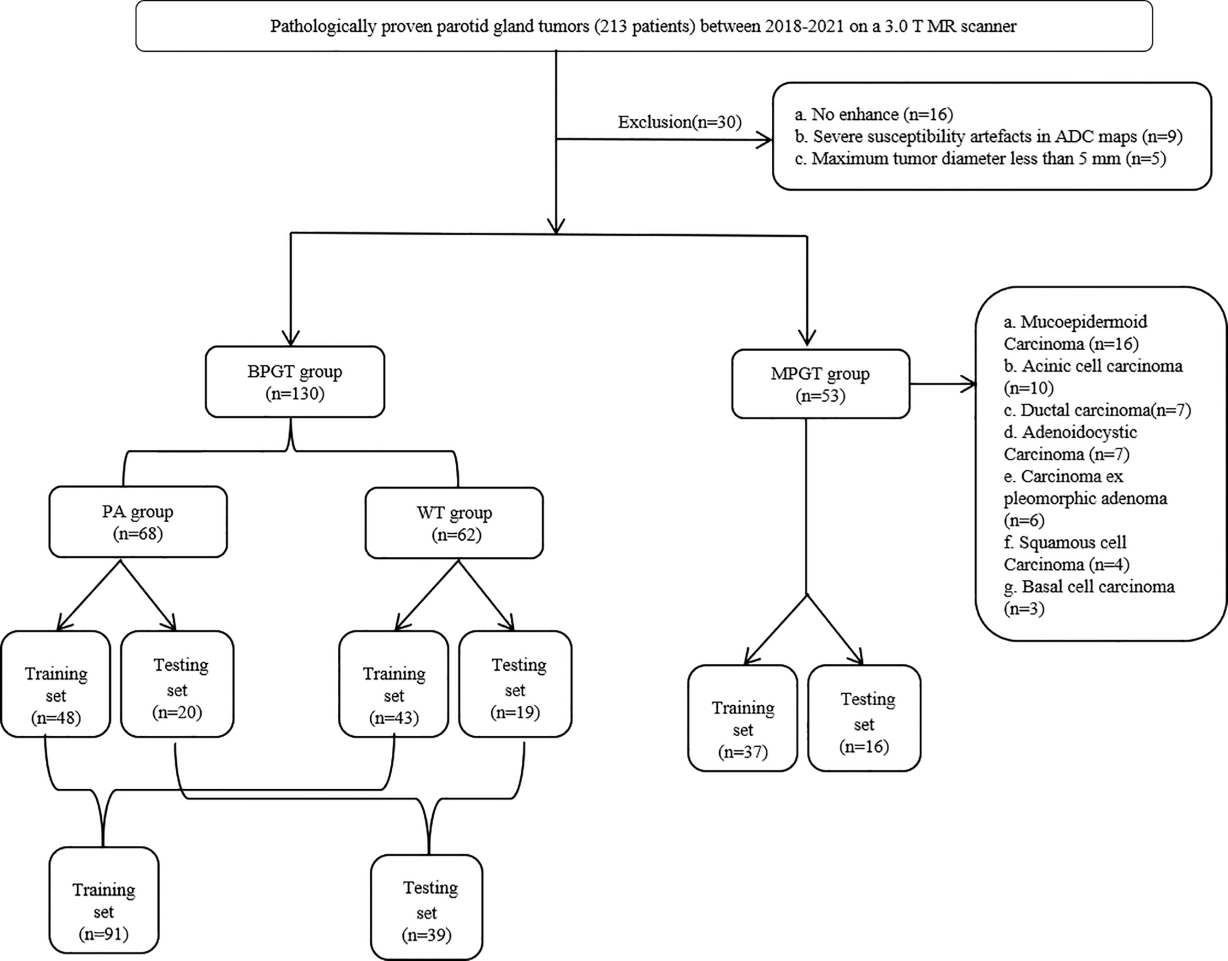
The flow chart of patient recruitment. PA, pleomorphic adenomas; WT, Warthin tumor; BPGT, benign parotid gland tumor, MPGT, malignant parotid gland tumor.

This study was approved by the review committee of the First Affiliated Hospital of Zhengzhou University (No: 2019-KY-0015-002). The requirement for informed consent was waived owing to the retrospective nature of this study. Between January 2018 and October 2021, 213 patients with a histological diagnosis of parotid tumors in surgically resected specimens underwent MRI. Parotid tumors were divided into four groups based on the pathological results, namely PA, WT, BPGT, and MPGT. The inclusion criteria were as follows: (a) patients with histologically confirmed parotid tumors and complete clinical data, and (b) patients who had undergone MR examination that included axial FS-T2WI, ADC and CE-T1WI less than 7 days before treatment. The exclusion criteria were as follows: (a) patients with a maximum tumor diameter of <5 mm to avoid bias due to partial volume effects, (b) presence of severe susceptibility artifacts or motion artifacts, and (c) absence of enhancement sequence. A total of 183 patients were randomly assigned to the training and test cohorts in a ratio of 7:3. A flow diagram of the study population is shown in **Figure 1**.

### MRI Data Acquisition

All images were taken using a 3.0 T MR scanner (Siemens Skyra, Prisma, Verio) with a 16-channel head and neck coil. The scan parameters were as follows: axial FS-T2WI, repetition time (TR)= 4000 ms, echo time (TE) = 83 ms, matrix = 320×224, slice thickness = 4.0 mm, and field of view (FOV) = 230 mm×230 mm; diffusion-weighted imaging (DWI), TR = 3300 ms, TE = 54 ms, matrix = 160×160, slice thickness = 4.0 mm, FOV = 240 mm×240 mm, b-value, 0, and 1,000 s/mm^2^ in three orthogonal directions; ADC maps were reconstructed automatically after DWI; axial CE-T1WI was performed following intravenous injection of 0.2 mL/kg of gadodiamide medium using a high-pressure syringe, followed by a 20 mL saline flush at the same injection rate, TR = 776 ms, TE = 10ms, matrix = 320×224, slice thickness = 4.0 mm, FOV = 230 mm×230 mm.

### MRI Morphological Feature Evaluation

Two radiologists with 10 and 7 years of experience in head and neck radiology who were blinded to the histopathological results evaluated the MRI features by consensus. The MRI features were as follows: (a) tumor margin (well-demarcated or poorly demarcated); (b) deep lobe involvement (DLI, absent or present; the superficial and deep lobes were divided by a line drawn from the lateral edge of the mandible to the lateral border of the posterior belly of the digastric muscle and retromandibular vein); (c) heterogeneous appearance (absent or present; 10% of the tumor has a different signal) (17); (d) cystic or necrotic regions (absent or present; an area with hyperintensity on FS-T2WI and no enhancement on CE-T1WI); (e) infiltration of surrounding tissue (IST, extension into the adjacent muscle group, subcutaneous space, and adjacent bone); and (f) contrast enhancement type (focal or diffuse).

### Image Segmentation and Radiomics Feature Extraction

The region of interest (ROI) was annotated manually from FS-T2WI using ITK-SNAP software (http://www.itksnap.org/pmwiki/pmwiki.php?n=Downloads.SNAP3) by two radiologists with 10 and 7 years of experience in head and neck radiology who contoured the outer edge of the tumor slice-by-slice. After which FS-T2WI was aligned onto the ADC maps and CE-T1WI, respectively. As the results of the radiomics feature calculation depend on the contours of ROIs delineated by radiologists, the intra-group correlation coefficient (ICC) was applied to assess the agreement of radiomics features extracted from these ROIs.

A total of 2553 (851 × 3) radiomics features were extracted from the ADC maps, FS-T2WI, and CE-T1WI using open-source software Feature Explorer (FAE, V 0.4.2). The radiomics features for each MRI sequence were as follows: shape (14 features); first-order statistics (18 features); and second-order features, including the gray level dependence matrix (14 features), gray-level co-occurrence matrix (GLCM, 24 features), gray level run length matrix (16 features), gray level size zone matrix (16 features), and neighborhood gray tone difference matrix (5 features). We also extracted 744 related features from the wavelet-transform images. Radiomics features with an ICC of >0.75 remained for the following analysis.

### Feature Preprocessing, Selection and Radiomics Model Construction

**Figure 2:**
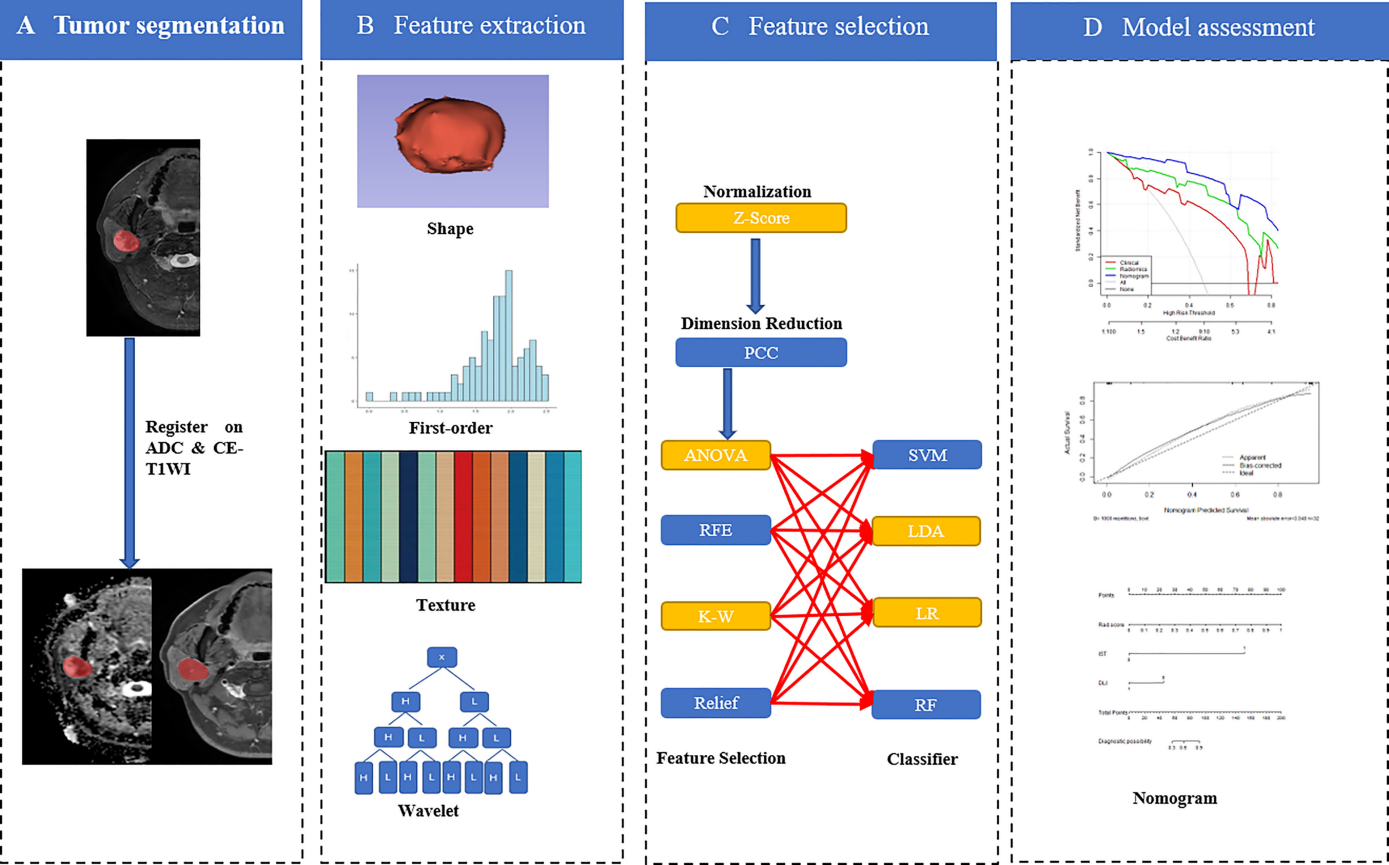
Workflow of the radiomics nomogram (order by A→D).

Up-sampling was performed to remove the imbalance of the datasets by repeating random cases to balance positive- and negative samples. Z-score normalization was performed as a pre-processing step on the feature matrix. Dimension reduction of the features was conducted using the Pearson correlation coefficient (PCC > 0.90) to reduce the feature matrix’s dimensions. Then four methods, namely analysis of variance, Kruskal-Wallis test, recursive feature elimination, and relief, were used to select the candidate features. Four classifiers, namely the support vector machine, linear discriminative analysis, logistical regression (LR), and random forest plots, were used to build single-, double-, and multi-sequence combined radiomics models. Five-fold cross-validation was applied to the training cohort to determine the candidate combinations of the selected features and classifiers. The cross-validation was then performed on the entire training cohort to determine the candidate combination, following which the final model was built by all training cases and evaluated on the independent test cohort. All the above were implemented in the open-source software Feature Explorer (FAE, V 0.4.2) (19). A flowchart describing the radiomic method is shown in **Figure 2**.

### Development of the Clinical Model and Radiomics Nomogram

Clinical features, such as sex, age, tumor margin, tumor location, IST, signal characteristics, and type of contrast enhancement were established in clinical models. The process for building the above models was similar to that used in radiomics model construction; however, the classifier used to construct clinical models only used the LR method to select features.

A radiomics score (Rad-score) was calculated for each patient using a linear combination of selected features that were weighted by their respective coefficients. The significant variables of the clinical factors and Rad-scores were integrated to build the combined model. The combined model was visualized as a radiomics nomogram to provide clinicians and patients with an individualized and easy-to-use tool for the preoperative prediction of parotid gland tumors.

### Performance Evaluation of the Models

The performances of these models were evaluated in the testing cohort using the receiver operating characteristic (ROC) curve and area under the curve (AUC). The accuracy, sensitivity, specificity, positive predictive value (PPV), and negative predictive value (NPV) were calculated to quantify discriminative performance. Calibration curves were used to graphically investigate the models’ performance characteristics. Three decision curve analyses (DCA), which were based on the clinical model, radiomics model, and radiomics nomogram, respectively, were used to assess the clinical usefulness of these models.

### Statistical Analyses

Statistical analyses of the clinical characteristics were conducted using IBM SPSS Statistics for Windows, version 26.0 (IBM Corp., Armonk, NY, USA). The normality of the distribution and homogeneity of the variance was evaluated using the Shapiro–Wilk and Levene’s tests, respectively. Continuous variables were compared using independent t-tests or Wilcoxon rank-sum tests, whereas categorical variables were compared using the chi-square test. Statistical significance was set at p<0.05.

## Results

### Clinical Factors and Performance of the Clinical Model

The demographic and MRI features of the patients are presented in **Table 1**. WTs are more common in older men than PA and MPGT. Significant differences were noted in differentiating BPGT from MPGT in terms of tumor margin, DLI, heterogeneous appearance, cystic or necrotic areas, IST, and type of contrast enhancement (*P* < 0.05). However, the above features did not differ significantly between PA and WT (*P* > 0.05). In clinical models, DLI and IST make significant contributions in differentiating BPGT from MPGT. Among the differences between PA and WT, age, and sex make significant contributions.

**Table 1:** Patient demographics and clinical information.

Clinical factors	Testing cohort (n=55)				*P*1	*P*2	*P*3	*P*4	Training cohort (n=128)				*P*1	*P*2	*P*3	*P*4
	PA (n=20)	WT (n=19)	BPGT (n=39)	MPGT (n=16)					PA (n=48)	WT (n=43)	BPGT (n=91)	MPGT (n=37)				
Age	32.53 ± 12.20	60.69 ± 8.82	47.67 ± 16.82	54.67 ± 13.84	0.000	0.000	0.425	0.667	37.50 ± 15.71	56.67 ± 15.48	47.11 ± 17.81	49.65 ± 15.00	0.000	0.050	0.077	0.470
Gender (M/F)	6/14	18/1	24/15	11/5	0.000	0.042	0.073	0.761	18/30	42/1	60/31	27/10	0.000	0.002	0.002	0.533
Margin (well-demarcated/poorly demarcated)	18/2	13/6	31/8	7/9	0.127	0.004	0.182	0.022	44/4	35/8	79/12	14/23	0.216	0.000	0.000	0.000
DLI (absent/present)	15/5	12/7	27/12	3/13	0.501	0.002	0.016	0.001	38/10	31/12	69/22	13/24	0.470	0.000	0.001	0.000
Heterogeneous appearance (absent/present)	10/10	13/6	23/16	4/12	0.333	0.176	0.018	0.037	32/16	21/22	53/38	9/28	0.094	0.000	0.037	0.001
Cystic or necrotic areas (absent/present)	15/5	12/7	27/12	5/11	0.501	0.017	0.092	0.015	30/18	22/21	52/39	13/24	0.297	0.016	0.179	0.032
IST (absent/present)	20/0	19/0	39/0	9/7	—	0.001	0.002	0.002	48/0	42/1	90/1	21/16	0.473	0.000	0.000	0.000
Type of contrast enhancement (focal/diffuse)	19/1	17/2	36/3	10/6	0.605	0.030	0.105	0.013	45/3	39/4	84/7	28/9	0.703	0.027	0.126	0.017

Numerical data are presented as mean ± standard deviation, categorical data as numbers (n). PA, pleomorphic adenomas; WT, Warthin tumor; BPGT, benign parotid gland tumor, MPGT, malignant parotid gland tumor; M, male; F, female; DLI, deep lobe involved; IST, infiltration of surrounding tissue; P1 Value, represents PA compared with WT; P2 Value, represents PA compared with MPGT; P3 Value, represents WT compared with MPGT; P4 Value, represents BPGT compared with MPGT. P-values of age and gender are the results of independent-samples t-tests; P-values of margin, DLI, heterogeneous appearance, cystic or necrotic areas, IST and type of contrast enhancement are the results of chi-square test.

### Performance of the Radiomics Models

**Figure 3:**
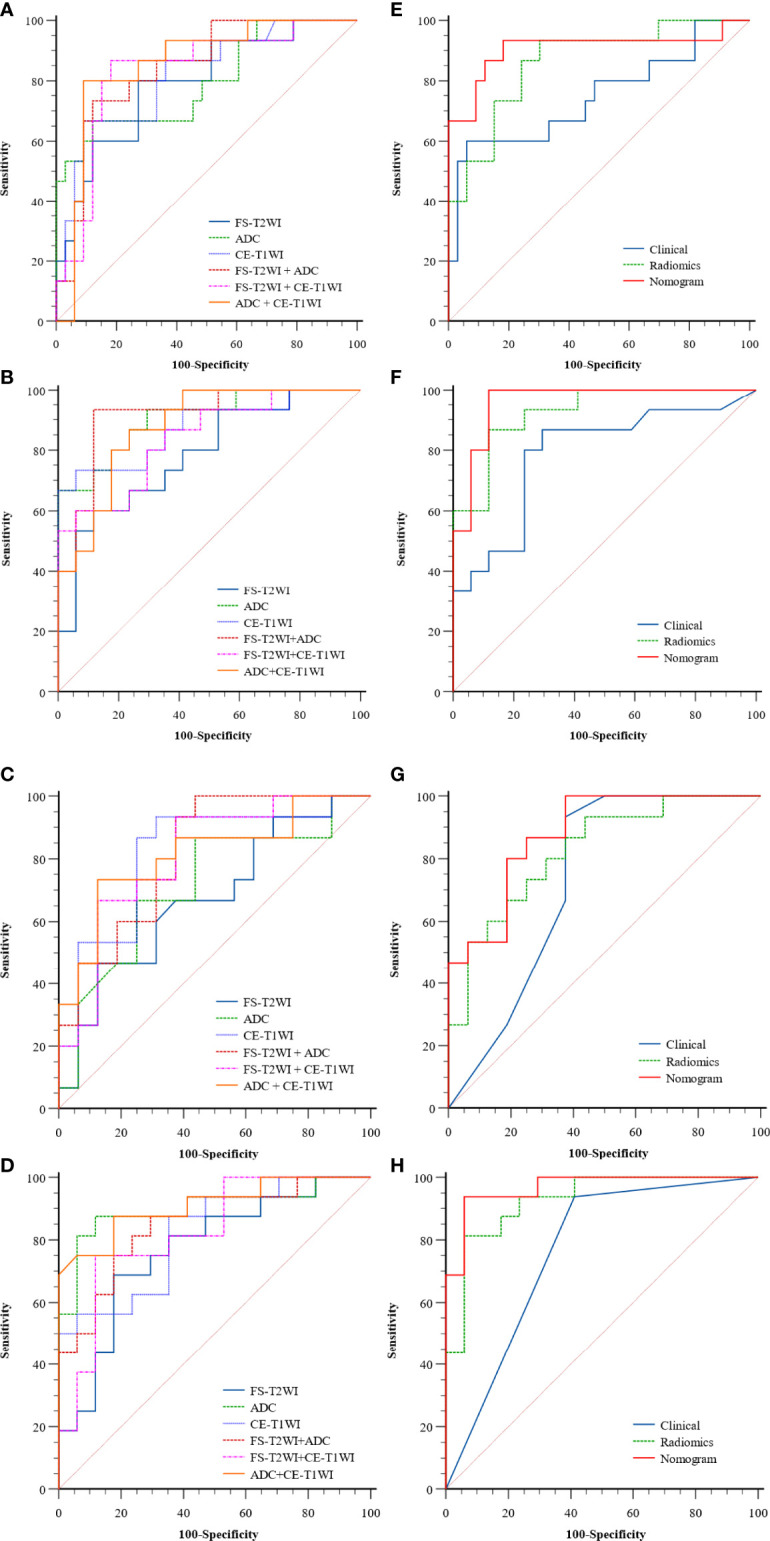
The ROC curves of the clinical model, radiomics models of FS-T2WI, ADC, CE-T1WI, FS-T2WI +ADC, FS-T2WI + CE-T1WI, ADC+ CE-T1WI **(A–D)** and, clinical, radiomics (FS-T2WI +ADC+ CE-T1WI), nomogram **(E–H)** in distinguishing parotid tumors of four groups: **(A, E)** BPGT *vs*. MPGT; **(B, F)** PA *vs*. MPGT; **(C, G)** WT *vs*. MPGT; and **(D, H)** PA *vs*. WT.

In the FS-T2WI, ADC, CE-T1WI, FS-T2WI+ADC, FS-T2WI+CE-T1WI, ADC+CE-T1WI, and FS-T2WI+ADC+CE-T1WI radiomics models, the multisequence combined radiomics model yielded the largest AUC (BPGT *vs*. MPGT, AUC=0.863; PA *vs*. MPGT, AUC=0.929; WT *vs*. MPGT, AUC=0.825; PA *vs*. WT, AUC=0.927) (**Figure 3**). The accuracy, sensitivity, specificity, PPV, and NPV of the radiomics models are shown in **Table 2**. The multisequence radiomics model was selected as the final radiomics model because of its improved performance; its selected radiomics features are presented in **Table 3**.

**Table 2:** The performance of the clinical models, radiomics models, and radiomics nomogram.

Model	AUC (95%*CI)*	Accuracy	Sensitivity	Specificity	PPV	NPV
**BPGT *vs*. MPGT**						
Clinical	0.748 (0.565-0.910)	0.833	0.600	0.939	0.818	0.838
FS-T2WI	0.792 (0.641-0.915)	0.750	0.800	0.727	0.571	0.889
ADC	0.796 (0.644-0.927)	0.813	0.667	0.879	0.714	0.853
CE-T1WI	0.817 (0.672-0.937)	0.833	0.667	0.909	0.769	0.857
FS-T2WI+ ADC	0.842 (0.710-0.948)	0.833	0.733	0.879	0.733	0.879
FS-T2WI+ CE-T1WI	0.830 (0.686-0.948)	0.792	0.867	0.758	0.679	0.926
ADC+ CE-T1WI	0.855 (0.733-0.960)	0.845	0.800	0.909	0.800	0.909
FS-T2WI+ ADC+ CE-T1WI	0.863 (0.735-0.963)	0.849	0.933	0.697	0.583	0.958
Nomogram	0.907 (0.765-0.993)	0.854	0.933	0.818	0.700	0.964
**PA *vs*. MPGT**						
Clinical	0.783 (0.591-0.937)	0.781	0.867	0.706	0.722	0.857
FS-T2WI	0.784 (0.600-0.925)	0.750	0.600	0.882	0.818	0.714
ADC	0.906 (0.767-0.990)	0.844	0.667	1.0	1.0	0.772
CE-T1WI	0.875 (0.732-0.980)	0.844	0.733	0.941	0.917	0.800
FS-T2WI+ ADC	0.914 (0.794-1.0)	0.906	0.933	0.882	0.875	0.938
FS-T2WI+ CE-T1WI	0.839 (0.691-0.952)	0.781	0.600	0.941	0.900	0.727
ADC+ CE-T1WI	0.878 (0.745-0.977)	0.813	0.867	0.765	0.765	0.867
FS-T2WI+ ADC+ CE-T1WI	0.929 (0.829-0.992)	0.875	0.867	0.882	0.867	0.882
Nomogram	0.961 (0.883-1.0)	0.938	0.902	0.882	0.882	0.948
**WT *vs*. MPGT**						
Clinical	0.708 (0.494-0.901)	0.807	1.0	0.625	0.714	1.0
FS-T2WI	0.673 (0.466-0.859)	0.677	0.467	0.875	0.778	0.636
ADC	0.713 (0.504-0.891)	0.710	0.867	0.563	0.650	0.818
CE-T1WI	0.817 (0.635-0.958)	0.807	0.933	0.688	0.737	0.917
FS-T2WI+ ADC	0.816 (0.652-0.963)	0.818	0.750	0.882	0.857	0.790
FS-T2WI+ CE-T1WI	0.808 (0.640-0.941)	0.774	0.933	0.625	0.700	0.909
ADC+ CE-T1WI	0.813 (0.638-0.949)	0.807	0.733	0.875	0.846	0.778
FS-T2WI+ ADC+ CE-T1WI	0.825 (0.663-0.954)	0.7419	0.933	0.563	0.667	0.900
Nomogram	0.879 (0.746-0.978)	0.807	1.0	0.625	0.714	1.0
**PA *vs*. WT**						
Clinical	0.763 (0.618-0.886)	0.758	0.938	0.588	0.682	0.909
FS-T2WI	0.768 (0.596-0.927)	0.758	0.688	0.824	0.786	0.737
ADC	0.901 (0.770-1.0)	0.879	0.875	0.882	0.875	0.882
CE-T1WI	0.820 (0.643-0.937)	0.758	0.875	0.647	0.700	0.846
FS-T2WI+ ADC	0.853 (0.722-0.967)	0.788	0.875	0.706	0.737	0.857
FS-T2WI+ CE-T1WI	0.824 (0.665-0.950)	0.818	0.750	0.882	0.851	0.790
ADC+ CE-T1WI	0.910 (0.794-0.993)	0.849	0.875	0.824	0.824	0.875
FS-T2WI+ ADC + CE-T1WI	0.927 (0.824-0.993)	0879	0.813	0.941	0.929	0.842
Nomogram	0.967 (0.897-1.0)	0.939	0.938	0.941	0.938	0.941

AUC, area under the curve; PPV, positive predictive value; NPV, negative predictive value; Vs, versus; PA, pleomorphic adenomas; WT, Warthin tumor; BPGT, benign parotid gland tumor, MPGT, malignant parotid gland tumor; FS-T2WI, fat-saturated T2-weighted image, CE-T1WI: contrast-enhanced T1-weighted image. The model of FS-T2WI+ ADC+ CE-T1WI was selected as the final radiomics model to build radiomics nomogram.

**Table 3:** Selected features and the coefficients of features in final radiomics model.

Features	Coefficients in model
**BPGT *vs*. MPGT**	
CE-T1WI_wavelet-HLL_ GLCM_ autocorrelation	2.233
ADC_ wavelet-LHL_ GLCM_ cluster shade	1.698
CE-T1WI_ wavelet-HLL_ NGTDM_ complexity	-0.355
FS-T2WI_ wavelet-HLL_ GLDM_ small dependence emphasis	-0.422
ADC_ original_ shape_ sphericity	-0.499
ADC_ wavelet-LHH_ GLCM_ mcc	-1.488
**PA *vs*. MPGT**	
ADC_ wavelet-LHL_ GLCM_ cluster shade	2.890
FS-T2WI_ wavelet-HLH_ GLSZM_ size zone non-uniformity normalized	1.620
CE-T1WI_ wavelet-HLL_ GLCM_ autocorrelation	1.388
ADC_ wavelet-LLH_ GLCM_ correlation	-0.135
ADC_ wavelet-LHL_ first-order_ skewness	-0.342
CE-T1WI_ wavelet-HLL_ GLSZM_ zone entropy	-0.881
ADC_ original_ shape_ sphericity	-3.566
**WT *vs*. MPGT**	
ADC_ wavelet-HHH_ GLSZM_ zone variance	1.512
CE-T1WI_ wavelet-HLH_ GLRLM_ run-variance	1.033
ADC_ wavelet-HHH_ GLSZM_ large area emphasis	0.223
FS-T2WI_ wavelet-HHH_ GLSZM_ gray level non-uniformity	-1.020
**PA *vs*. WT**	
ADC_ wavelet-LHL_ first-order_ median	3.509
CE-T1WI_ wavelet-LLH_ first-order_ kurtosis	1.102
FS-T2WI_ wavelet-LLL_ first-order_ skewness	0.920
ADC_ wavelet-HLH_ GLCM_ correlation	0.535
CE-T1WI_ wavelet-LLH_ GLCM_ idn	0.340
ADC_ original_ first-order_ 10percentile	-0.531
FS-T2WI_ wavelet-HHL_ GLCM_ small dependence high gray level emphasis	-1.413
ADC_ wavelet HHH_ GLSZM_ size zone non-uniformity normalized	-1.504

GLCM, gray-level co-occurrence matrix; GLDM, gray-level dependence matrix; GLRLM, gray-level run length matrix; GLSZM, gray-level size zone matrix; Vs, versus; PA, pleomorphic adenomas; WT, Warthin tumor; BPGT, benign parotid gland tumor, MPGT, malignant parotid gland tumor; FS-T2WI, fat-saturated T2-weighted image, CE-T1WI: contrast-enhanced T1-weighted image.

### Performance of the Radiomics Nomogram Model

**Figure 4:**
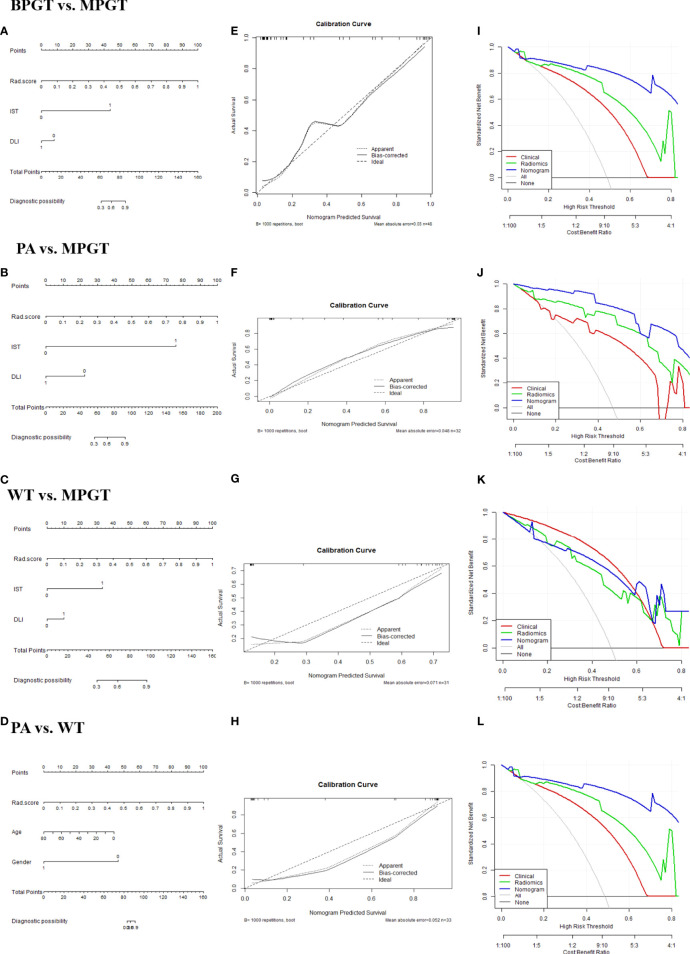
The radiomics nomogram **(A–D)**. Calibration curves **(E–H)**. The dotted diagonal line represents an ideal evaluation, while the solid lines represent the performance of the nomogram. Closer to the dotted diagonal line indicates better evaluation. DCA curves **(I–L)** of the clinical, radiomics (equals FS-T2WI +ADC+ CE-T1WI model), and nomogram model.

The radiomics nomogram was constructed by incorporating a multi-sequence combined radiomics model, DLI, and IST to differentiate BPGT from MPGT, and differentiate PA and WT from MPGT. In the multi-sequence combined model, age and sex were integrated to build a radiomics nomogram to differentiate PA from WT (**Figures 4A–D**). The radiomics nomogram model achieved the largest AUC compared with the clinical and radiomics models (**Table 2**). In addition, the nomogram yielded better diagnostic performance in differentiating BPGT from MPGT (AUC=0.907), and PA from MPGT (AUC=0.961), compared with differentiating WT from MPGT (AUC=0.879). DeLong’s test revealed that the AUCs of the clinical model and nomogram differed significantly between all groups in both the training and testing cohorts (p < 0.05). Although the AUC of the nomogram was higher than that of the radiomics models, the results of DeLong’s test showed no significant difference in differential diagnostic performance between the two groups (**Table 4**). The calibration curves of the nomogram based on the four groups showed good calibration in the testing cohorts (**Figures 4E–H**). The DCAs of the three models are shown in **Figures 4I–L**.

**Table 4:** Comparison of the performance of the models.

Comparison	DeLong’s test* (p-value) in the testing cohort	DeLong’s test* (p-value) training cohorts
BPGT *vs*. MPGT		
Clinical model *vs*. radiomics model	0.404	0.103
Clinical model *vs*. radiomics nomogram	0.041	0.010
Radiomics model *vs*. radiomics nomogram	0.406	0.172
PA *vs*. MPGT		
Clinical model *vs*. radiomics model	0.163	0.009
Clinical model *vs*. radiomics nomogram	0.048	0.013
Radiomics model *vs*. radiomics nomogram	0.434	0.100
WT *vs*. MPGT		
Clinical model *vs*. radiomics model	0.359	0.046
Clinical model *vs*. radiomics nomogram	0.016	0.013
Radiomics model *vs*. radiomics nomogram	0.603	0.616
PA *vs*. WT		
Clinical model *vs*. radiomics model	0.034	0.001
Clinical model *vs*. radiomics nomogram	0.006	0.000
Radiomics model *vs*. radiomics nomogram	0.395	0.519

p-value < 0.05 indicated a statistically significant difference. *Test for the comparison of the difference of AUC; Vs, versus; PA, pleomorphic adenomas; WT, Warthin tumor; BPGT, benign parotid gland tumor, MPGT, malignant parotid gland tumor.

## Discussion

In our study, the radiomics model based on multi-sequence MRI exhibited improved performance when differentiating BPGT from MPGT, and PA from WT preoperatively. The radiomics nomogram that incorporated both radiomics and clinical features was superior to the clinical model, which demonstrated the incremental value of the radiomics model when differentiating parotid tumors. Furthermore, the nomogram yielded better diagnostic performance in differentiating BPGT from MPGT, and PA from MPGT, compared with differentiating WT from MPGT.

A radiomics model based on multi-sequence MRI can provide comprehensive information related to tumor heterogeneity (20–22). In our study, we found that the multi-sequence combined radiomics model exhibited better diagnostic performance than single- and double-sequence radiomics models when differentiating parotid tumors. The performance of the radiomics model worsened when differentiating BPGT from MPGT using single- and double-sequence radiomics models due to the overlap in ADC values between WT and MPGT (23). Meanwhile, BPGT also showed signs of enhancement compared with MPGT (24). Given the complexity of tumor components, the signal intensity in FS-T2WI between BPGT and MPGT might overlap, resulting in low specificity (9, 16). FS-T2WI can provide anatomical information on the tumors while ADC maps reflect information on the architecture and density of tumor cells, and CE-T1WI provides information of the tumor local blood supply (8–10). The combination of these three sequences can reflect the biological information of parotid gland tumors more comprehensively.

Adequate clinical information and radiological characteristics facilitate accurate distinction between BPGT and MPGT. Reportedly, BPGTs are primarily located in the superficial lobe, whereas MPGTs tend to arise in the deep lobe or both lobes (1, 25). In the present study, we found that poorly demarcated margins, a heterogeneous appearance, and cystic degeneration or necrosis were more common in MPGT than in BPGT, which is consistent with the findings of previous studies (26, 27). The presence of IST is also indicative strongly of malignancy (28). Moreover, the features of DLI and IST were selected to build the nomogram, which was consistent with previous studies (17).

The features of the transform wavelet account for a relatively large proportion of the final radiomics model for differentiating parotid gland tumors. We speculate that transform-wavelet higher-order statistics may highlight details in the original images and further reflect the heterogeneity between tumors (29, 30); therefore, they can extract increasingly coarse features in a more flexible manner. Consistent with previous studies (31, 32), we found that the features of autocorrelation and cluster shade from GLCM were selected to differentiate BPGT from MPGT, as well as PA from MPGT. The coefficients of these features in the final radiomic model were relatively large. This could be explained by the higher tissue heterogeneity in MPGT compared with BPGT (33, 34). In fact, the structural components of malignant tumors are more commonly mixed with bleeding and necrosis, leading to greater asymmetry and lower autocorrelation compared with benign tumors with a more regular and homogeneous structure. Nevertheless, the difference is that our study included clinical variables that achieved a higher AUC than the radiomics and clinical models, which greatly compensates for previous studies and will accelerate its clinical application.

Although we successfully built a radiomics nomogram that achieved good results in differentiating BPGT from MPGT, and PA from MPGT, the reliability and accuracy of differentiating WT from MPGT were relatively low. WT may have relatively greater tissue heterogeneity and vascular distribution than PA, making it more similar to low-grade malignant tumors (27, 34, 35). This may also explain the greater reliability and accuracy in differentiating PA from MPGT compared with BPGT and MPGT.

For differentiation of PA from WT, our study found that the radiomics nomogram incorporating radiomics features, age, and sex exhibited slightly better results than the radiomics nomogram used by Zheng et al. (36). Radiomics feature-based ADC also plays an important role in the final radiomics model. This may be because PA is rich in myxoid and chondroid tissue stroma and has a larger extracellular space than WT, causing a considerable difference in heterogeneity in the ADC model (37, 38). This also verifies the application of ADC values in the quantitative analysis to some extent to differentiate PA from WT (39). FS-T2WI or CE-T1WI combined with ADC can improve the performance of the single-sequence model, consistent with previous studies (17, 40, 41).

The present study had some limitations. First, the retrospective design of this study may cause selection bias. Second, advanced sequences proven useful in identifying parotid gland tumors in previous studies were not included, such as intravoxel incoherent motion and diffusion kurtosis imaging. Third, this study was a single-center study. Additional patients from other centers are required as an external validation dataset to validate these models and improve their universality and stability.

## Conclusion

Radiomics nomogram incorporates radiomics and clinical features to differentiate BPGT from MPGT, PA from MPGT, and PA from WT exhibited excellent diagnostic performance. It also exhibited good diagnostic performance in differentiating WT from MPGT, which can serve as a valuable clinical tool for clinical decision-making.

## Data Availability Statement

The raw data supporting the conclusions of this article will be made available by the authors, without undue reservation.

## Ethics Statement

This study was approved by the review committee of the First Affiliated Hospital of Zhengzhou University (No: 2019-KY-0015-002). Written informed consent for participation was not required for this study in accordance with the national legislation and the institutional requirements.

## Author Contributions

JQ and XM made contribution to collecting patients. JQ, AG, GZ, YS, YZ, and JC made data analysis and interpretation. JQ, JC, XM, and AG were major contributors. All authors made a substantial contribution to researching data, discussion of content, reviewing and editing manuscript before submission. All authors read and approved the final manuscript.

## Funding

This study was supported by a public service platform for artificial intelligence screening and auxiliary diagnosis for the medical and health industry, Ministry of Industry and Information Technology of the People’s Republic of China (No. CEIEC-2020-ZM02-0103/03); the joint construction project of Henan medical science and technology research project (No. LHGJ20190157) and the youth project of Henan medical science and technology research project (No. SBGJ202103078).

## Conflict of Interest

Author YS was employed by the company Siemens Healthineers Ltd.

The remaining authors declare that the research was conducted in the absence of any commercial or financial relationships that could be construed as a potential conflict of interest.

## Publisher’s Note

All claims expressed in this article are solely those of the authors and do not necessarily represent those of their affiliated organizations, or those of the publisher, the editors and the reviewers. Any product that may be evaluated in this article, or claim that may be made by its manufacturer, is not guaranteed or endorsed by the publisher.

## Abbreviations

BPGT, benign parotid gland tumor; MPGT, malignant parotid gland tumor; PA, pleomorphic adenoma; WT, warthin tumor; Rad-score, radiomics score; AUC, area under the curve; FNA, fine-needle aspiration; MRI, magnetic resonance imaging; ADC, apparent diffusion coefficient; DWI, diffusion-weighted imaging; T2WI, T2-weighted image; FS-T2WI, fat-saturated T2-weighted image; CE-T1WI, contrast-enhanced T1-weighted image; TR, repetition time; TE, echo time; FOV, field of view; ROI, the region of interest; ICC, intra-group correlation coefficient; DLI, deep lobe involvement; IST, infiltration of surrounding tissue; GLCM, gray level co-occurrence matrix; GLDM, gray level dependence matrix; GLRLM, gray level run length matrix; GLSZM, gray level size zone matrix; NGTDM, neighborhood gray tone difference matrix; PCC, Pearson correlation coefficient; LR, logistic regression; ROC curve, receiver operating characteristic curve; PPV, positive predictive value; NPV, negative predictive value; DCA, decision curve analysis.
